# Natural Products Improving Hyperuricemia with Hepatorenal Dual Effects

**DOI:** 10.1155/2016/7390504

**Published:** 2016-10-25

**Authors:** Shijun Hao, Chunlei Zhang, Haiyan Song

**Affiliations:** ^1^School of Public Health, Shanghai University of Traditional Chinese Medicine, Shanghai 201203, China; ^2^Institute of Digestive Diseases, Longhua Hospital, Shanghai University of Traditional Chinese Medicine, Shanghai 200032, China

## Abstract

This review aims to put forth an overview of natural products reducing uric acid level with hepatorenal dual effects. The prevalence of hyperuricemia increased rapidly in recent years and has closely interdependent relationship with other metabolic disorders. Current therapeutically used drugs including a few uricostatic and uricosuric chemical drugs are proved efficient to control serum uric acid level. However, their side effects as well as contraindication in some cases with liver, kidney injury, or other conditions frequently limit their clinic application. More attention thus has been paid to natural products as an alternative means in treating hyperuricemia. Many natural products have been proved efficient in downregulating uric acid level, among which some can improve hyperuricemia with hepatorenal dual effects. It means these natural products can regulate both the production and the excretion of uric acid by targeting the key metabolic enzymes mainly in liver or uric acid transporters in kidneys. Thus, these natural products could have stronger efficacy and broader application, which may be developed for the treatment of hyperuricemia in clinic.

## 1. Introduction

Uric acid is the end item of purine metabolism in human body, originating from hypoxanthine after double enzyme catalysis by xanthine oxidase (XOD) [1]. Normally it has multiple physiological effects, including modulation of immune responses, regulation of blood pressure, and controlling anti-/prooxidative balance [2]. Either overgeneration of uric acid or a reduction in its excretion can lead to hyperuricemia. The risk factors of hyperuricemia include age, gender, race, genetic factors, environmental factors, and dietary habits. National Health and Nutrition Examination Survey 2007-2008 in USA and Taiwan Nutrition survey demonstrated that the serum uric acid (SUA) level increases with more intake of meat, seafood, and alcohol, especially beer correspondingly [3, 4]. In the past decades, with the change in diet, the prevalence of hyperuricemia has increased worldwide. The incidence of hyperuricemia is 21.2% and 21.6% among men and women, respectively, in USA [5] and 42.1% and 27.4% among men and women in Taiwan [6]. Hyperuricemia is predicted to be the second popular metabolic disease after type 2 diabetes in the future [7].

Uric acid is the well known primary risk factor for developing symptomatic gout [8]. Recently, high level of uric acid has been identified closely related to all the metabolic diseases, such as obesity, hypertension, type 2 diabetes, nonalcoholic fatty liver disease, coronary artery disease, and stroke, in addition to being involved in the pathogenesis of gout and chronic nephropathy [9–13]. It was reported that the prevalence of metabolic syndrome (MS) was high among patients with gout. Even, in those without gout, the prevalence of MS was more than 10-fold higher in those with uric acid levels of 10 mg/dL or greater compared with uric acid levels less than 6 mg/dL. Thus, higher uric acid levels are related to MS, and the prevalence of MS also increased significantly with uric acid levels [14].

## 2. The Mechanism Contributing to Hyperuricemia and Current Target Medicines

The production of uric acid is regulated by the endogenous (nucleotides originating from cellular metabolism) and exogenous (dietary) precursors transported to the liver, and the excretion is controlled by the kidneys through renal plasma flow, glomerular filtration, and proximal tubular exchange. Adenine nucleoside and guanine nucleoside can be catalyzed to generate hypoxanthine and guanine, respectively. Through the function of enzyme XOD or guaninase, hypoxanthine and guanine are converted to xanthine, which is subsequently catalyzed into the final product uric acid by XOD. The inhibitors of XOD are proved effective in patients who overproduce uric acid [29, 30]. XOD can be detected using high sensitive method of radioimmunoassay in many kinds of human tissues, but its activity in other tissues was only 1/10 to 1/1000 compared with that in liver tissue [31, 32].

**Figure 1:**
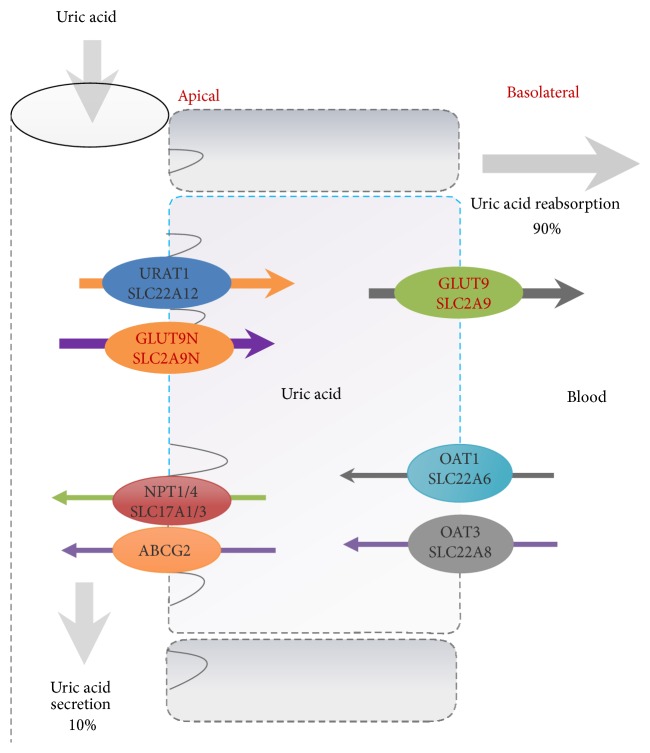
The processes of uric acid excretion and reabsorption and the main transporters in renal tubular epithelial cell.

It was reported that less excretion is the pivotal factor of primary hyperuricemia, accounting for about 90% of the cases [29]. Approximately two-thirds of the uric acid load is eliminated through kidneys, while the gastrointestinal tract eliminates one-third [33]. Thus, the kidney is another important organ for regulating uric acid level. Almost all uric acid is filtered from glomeruli, while postglomerular reabsorption and secretion regulate the amount of uric acid excretion. The proximal tubule is responsible for the reabsorption and secretion of uric acid, and approximately 90% is reabsorbed into blood [34]. Therefore, urate transport system of renal proximal tubules plays a vital role in the determination of serum urate levels [35]. It has been reported that urate anion exchanger 1 (URAT1/SLC22A12) and glucose transporter 9 (GLUT9/SLC2A9) play important roles in uric acid reabsorption. Similar to the reabsorption, uric acid secretion in human proximal tubules is performed mainly by exchangers organic anion transporter 1 (OAT1/SLC22A6) and/or OAT3 (SLC22A8) [36].  Figure 1 demonstrated the processes of uric acid secretion and reabsorption in renal proximal tubules as well as the transporters. Consequently, either overgeneration of uric acid (mainly in liver), as in MS or having diets rich in fructose and purines, or a reduction in its excretion (mainly in kidneys), as in acute renal failure or consequent to some drugs, can lead to high serum uric acid levels.

Nonpharmacological therapy including dietary poor in purine-rich food, sugars, alcohol, and rich in vegetables and water intake is necessary for hyperuricemia. However, it is not enough for patients with higher uric acid level. Pharmacological therapy is necessarily required. Since urate homeostasis depends on the balance mainly between production in liver, secretion, and reabsorption in kidney tubule and excretion in intestine, uric-acid-lowering drugs act on inhibiting generation, reducing absorption, and increasing secretion. Currently, the long-term treatment of hyperuricemia is aimed at modulating the activity of key enzymes involved in the metabolism and excretion of uric acid, like XOD and URAT1. Medicines are divided into two main classes: uricostatic drugs (e.g., allopurinol) and uricosuric drugs (e.g., sulphinpyrazone, probenecid, and benzbromarone) [37]. The target of uricostatic drugs focused primarily on XOD, for example, allopurinol, which inhibits XOD since it is an analogue of hypoxanthine. Allopurinol is the classical therapeutically used uricostatic drug and oxypurinol, tisorurinol, febuxostat, topiroxostat, and so forth have the same uricostatic effects. Since kidneys reabsorbed around 90% of filtered urate, inhibiting absorption is especially important in uricosuric drugs. The intestine is responsible for 30% of total body uric acid excretion. By far, the drug research for inhibiting intestinal excretion has been rare. The uricostatic and uricosuric drugs are proved efficient to control serum uric acid level; however, the side effects frequently restricted their clinic application. Among the patients treated by allopurinol, about 2% of patients appeared to have skin rash, 0.4% patients have kidney failure, or concomitant thiazide diuretic therapy may experience a severe idiosyncratic reaction, known as allopurinol hypersensitivity syndrome [38]. Uricosuric agents such as sulphinpyrazone and probenecid are relatively contraindicated in patients with kidney stone. Benzbromarone can be used in patients with chronic kidney disease but may cause incidental risk of hepatotoxicity [39].

## 3. Natural Products for Improving Hyperuricemia

During the past decades, more and more attention has been paid to natural products as alternative methods in treating hyperuricemia. Natural products reducing uric acid are also divided into two main classes: uricostatic and uricosuric drugs. There are a large number of Chinese medicines or the extracted compounds proved to be able to inhibit XOD activity to attenuate production of uric acid. Glabrous greenbrier rhizome, radix puerariae, mangiferin, celery, turmeric, motherwort, berberine, and so forth have been evaluated as active in inhibiting the enzyme XOD, from a total of 122 traditional Chinese medicines selected according to the clinical efficacy and prescription frequency for the treatment of gout and other hyperuricemia related disorders [40]. Relatively less studies reported natural medicines with effects on the renal urate transport system. Quercetin, at the dose of 50 and 100 mg/kg, could effectively upregulate OAT1 and downregulate GLUT9 and URAT1 in the kidneys of hyperuricemic mice [41]. Esculetin and esculin were found to improve hyperuricemia and renal dysfunction through upregulating OAT1. Through inhibiting GLUT9 or URAT1 in kidneys of hyperuricemic mice, fraxetin and fraxin could enhance urate excretion to some extent. Cortex fraxini coumarines were reported to be partly contributing to their functions of lower SUA level by regulating ABCG2 [42].

Among the reported drugs for treating hyperuricemia, we found some drugs playing the role through hepatorenal dual regulation, by targeting both XOD and excretion concurrently, which may enhance their function and broaden their application condition. The majority of these medicines belong to traditional Chinese medicine and their components. In this section, we focused on the natural products which have hepatorenal dual effects on hyperuricemia. To search for these natural products, we first used the following key words in PubMed: “uric acid” or “hyperuricemia” or “gout” and “medicinal plant” or “herb” or “herbal medicine” or “natural products” or “phytomedicine” or “phytotherapy” and “xanthine oxidase” or “XOD” or “XDH”. On this basis, we searched for renal urate transports separately, using words such as “uricosuric” or “URAT1” or “GLUT9” or “OAT1” or “OAT3” or “OAT4” or “ABCG2” or “NPT1” or “NPT4” or “MRP4” or “PDZK1”. In addition, we also searched for the aforementioned key words in Chinese from CNKI (China National Knowledge Infrastructure) and WANFANG DATA knowledge service platform. The results are listed in Table 1.

### 3.1. Natural Products with Effects of Inhibiting Generation and Absorption of Uric Acid (Target URAT1, GLUT9, and XOD)

In the kidney, the absorption of urate can be divided into two stages: urate is first absorbed from renal tubular cavity to renal tubular epithelial cells (mainly mediated by URAT1), subsequently, absorbed into blood across the tubular epithelial basement membrane (mainly mediated by GLUT9) [43]. The kidney specific urate transporter URAT1 (SLC22A12) was first identified by Enomoto et al. It was demonstrated that this molecule is the drug target that alters serum uric acid levels and causes idiopathic renal hypouricemia [44]. The URAT1 protein is specifically localized in the brush border membrane of the proximal tubule. It participates in the apical (luminal) uptake of urate from the primary urine to the proximal tubule cell, thus affecting reabsorption. The* in vivo* experiments found that URAT1 was a biological target of some uricosuric drugs, including probenecid, indomethacin, 6-hydroxybenzbromarone, and salicylate [45]. Li et al. first reported that GLUT9 (SLC2A9) is a gene correlated with SUA level [46]. Subsequently similar results have been reported by several other studies [47–52]. Human GLUT9 has two isoforms (GLUT9 and GLUT9N) depending on the splicing of the intracellular part of the N-terminal region [47]. When artificially expressed in polarized MDCK cells, GLUT9 (or long form) is expressed at the basal side, and GLUT9N (or short form) is expressed at the apical side. GLUT9N transports uric acid from renal tubular cavity into epithelial cells, whereas GLUT9 transits uric acid from epithelial cells into tubulointerstitium or blood [44]. Studies showed that GLUT9 plays a more important role than URAT1 in the absorption of uric acid [53].

In the* in vitro* research, Hou et al. showed that dried longan seed extract (LSE) and its active ingredients inhibited XOD in a dose dependent manner [15]. In the* in vivo* experiment, LSE was discovered to able to reduce serum XOD activity and SUA level in hyperuricemic rats. In addition, LSE increased GLUT1 but decreased GLUT9 protein level in kidney, respectively. These results showed that longan seeds were effective against hyperuricemia and indicated that its effect depended on inhibiting XOD and modulating urate transporters. Zeng et al. studied the effect of* Plantago asiatica* L. herbs extracts (PAHEs) on serum levels of uric acid level in hyperuricemia mice. The results demonstrated that PAHEs could obviously improve hyperuricemia. The mechanisms include downregulating hepatic ADA and XOD to reduce production of uric acid and enhancing urate excretion and decreasing urate reabsorption by suppressing renal URAT1 [16]. Bergenin belongs to isocoumarin compounds, which can protect liver, shrink ulcer, and improve immune functions [17], and has the inhibitory activity for XOD [54]. Zhou and Chen used bergenin to treat hyperuricemia model mice induced by potassium oxonate [18]. The results showed that bergenin significantly reduced SUA and creatinine level, and urea nitrogen in model mice improved the 24 h excretion of uric acid and creatinine. Bergenin obviously lowered the expression of URAT1 and GLUT9 in kidneys of model mice. The experiments of Xu showed that sea cucumber saponin and polysaccharides from* Pearsonothuria graeffei* (Pg),* Apostichopus japonicus* (Aj),* Cucumaria frondosa* (Cf), and* Isostichopus badionotus* (Ib) could significantly lower SUA level in hyperuricemia mice induced by feeding yeast extract powder for 14 days, and all of the natural products could inhibit hepatic XOD and adenylate deaminase (ADA) activities. Furthermore, the sea cucumber saponin and polysaccharides were found to downregulate the mRNA levels of hepatic ADA and XOD and renal GLUT9, which is the important transporter in the process of the reabsorption of uric acid [19]. Shi et al. explored the effect of puerarin on SUA content and pathways in hyperuricemic rats. This research monitored indicators of hyperuricemic rats intervened with puerarin, such as SUA, oxidase activity of XOD, and uric acid excretion. The results showed that puerarin at reasonable dosage was beneficial to improve SUA levels via inhibiting activity of XOD and promoting uric acid excretion [20].

### 3.2. Natural Products with Effects of Inhibiting Generation and Promoting Secretion of Uric Acid (Target OAT1, OAT3, and XOD)

The organic anion and urate transporters OAT1 (SLC22A6) and OAT3 (SLC22A8) can act as urate/dicarboxylate exchangers [55–57] and are found on the basolateral side of the same cells that express OAT4 [58]. Gene knockout studies* in vivo* found that absence of OAT1 or OAT3 slightly decreased uricosuria, suggesting that their principal function is in urate excretion [59].

Zhou et al. studied the effect of saponins from Rhizoma Dioscoreae Nipponicae in hyperuricemia models and verify the anti-inflammatory effect of these saponins* in vitro*. In the hyperuricemia model mice, it was found that total saponins from Rhizoma Dioscoreae Nipponicae at different doses (600, 300, and 30 mg/kg) could significantly reduce uric acid level through inhibiting the activities of both ADA and XOD. Meanwhile, these saponins could upregulate the expression of OAT1 [21]. The study of Xu showed that the antihyperuricemia effect of saponin and nonsaponin residue was related to the inhibition of hepatic XOD and ADA activities. Dietary sea cucumber saponin-EA also could decrease serum uric acid level significantly by 18.1%. EA was found to increase the secretion of uric acid and upregulate the mRNA levels of renal organic anion transporter 1 (OAT1). Moreover, the expression and activity of hepatic XOD and ADA were elevated by EA [19].

### 3.3. Natural Products with Effects of Inhibiting Generation, Absorption, and Promoting Secretion of Uric Acid (Target OAT, GLUT9, and XOD)

Green tea polyphenols (GTP) are generally well known as the major active component with multiple pharmacological functions in green tea. In one study to investigate the effect of GTP on SUA level, it was found that GTP significantly decreased SUA levels in a dose dependent manner in potassium oxonate-induced hyperuricemic mice. Furthermore, GTP reduced XOD expression in liver and reduced URAT1 expression and increased OAT1 and OAT3 expressions in kidneys, suggesting that GTP might attenuate SUA level through decreasing production and increasing excretion of uric acid [22]. Hu et al. found Jasminoidin significantly lowered the SUA levels and increased the uric acid excretion. This compound from Chinese medicine remarkably inhibited the hepatic XOD activities and regulated the expressions of renal urate transporters in mice [23].* Smilax riparia* is a botanical widely grown in southern and central region of China. The roots and rhizomes of* Smilax riparia* in traditional Chinese medicine (TCM) have been used to treat the symptoms of gout and hyperuricemia related conditions, including inflammation and some malignancies [60, 61]. Studies by Hou et al. indicated that the synergistic effects of allopurinol combined with pallidifloside D were associated with the inhibition of both serum and hepatic XOD, upregulation of mOAT1, and downregulation of renal GLUT9 and URAT1 [24]. Zhu and Chen studied and found that the total saponins of* Dioscorea* can significantly decrease the level of SUA in hyperuricemia rats caused by adenine and ethambutol. The mechanism may be related to inhibiting XOD activity, as well as increasing the excretion of uric acid through the downregulation of high expression of URAT1 and upregulation of OAT1 and OAT3 [25]. Zeng et al. researched the influence of* Lagotis brevituba* maxim extracts (LBMs) on the level of uric acid in hyperuricemia mice [26]. The results demonstrated that LBMs possessed antigout effect. The main mechanism included inhibiting the activity of hepatic XOD, downregulating renal URAT1 and GLUT9, and upregulating OAT1 expression in hyperuricemiamice. Wang et al. found that* P. sibircum* Laxm. alcohol extract could distinctly lower the SUA level, and the mechanism is related to the inhibition of hepatic XOD activities and the regulation of renal urate transporters. In addition, alcohol extract of* P. sibircum* Laxm. significantly reduced the mRNA expression level of renal GLUT9 in mice and upregulated OAT1 mRNA expression. At the dose of 8.0 g/kg,* P. sibircum* Laxm. alcohol extract also significantly reduced URAT1 in mice [27].

Modified Simiao Decoction (MSD), a complex recipe of Chinese medicine, has been used in recent decades and proven to be efficient in treating gout and hyperuricemia. Hua et al. investigated the effects of MSD in hyperuricemic mice. MSD could decrease SUA levels, serum creatinine, and BUN and restrain XOD activities in liver and serum. It also upregulated OAT1 and downregulated URAT1 protein expressions in the renal tissues of hyperuricemic mice in dose dependent manner [28].

## 4. Conclusion

A lot of natural products have been proved efficient in downregulating uric acid level, among which some target both the production and the excretion of uric acid. In this case, the function is more powerful and the application condition of patients will have less limitation. Therefore, these natural products for treating hyperuricemia should be given more attention in the future. However, clinical studies for the therapeutic efficacy of these natural products and the underlying mechanism are still required.

## Figures and Tables

**Table 1:** The hepatorenal dual actions of natural products on hyperuricemia.

Natural products	Inhibition of the generation	Inhibition of the absorption	Promote the secretion	Refs.
Longan seed extract (LSE)	Inhibit activity of XOD/ADA	Decrease GLUT9 but increase GLUT1	None	[15]
*Plantago asiatica* L. herbs extracts (PAHEs)	Inhibit activity of XOD	Downregulate renal URAT1	None	[16]
Bergenin	Inhibit activity of XOD	Downregulate URAT1 and GLUT9	None	[17] [18]
Sea cucumber saponin	Inhibit activity of XOD/ADA	Downregulate GLUT9	None	[19]
Sea cucumber polysaccharides	Inhibit activity of XOD/ADA Downregulate XOD/ADA	Downregulate GLUT9	None	[19]
Puerarin	Inhibit activity of XOD	Unidentified	Unidentified	[20]
Total saponins from Rhizoma Dioscoreae Nipponicae	Inhibit activity of XOD/ADA	None	Upregulate OAT1	[21]
Sea cucumber saponin-EA	Inhibit activity of XOD/ADA	None	Upregulate OAT1	[19]
Jasminoidin	Inhibit activity of XOD	Regulate expressions of renal urate transporters	None	[22]
Green tea polyphenols	Reduce XOD expression	Reduce URAT1	Upregulate OAT1 and OAT3	[23]
*Smilax riparia* A. D. C	Inhibit activity of XOD	Downregulate renal URAT1 and GLUT9	Upregulate OAT1	[24]
Modified Simiao Decoction	Inhibit activity of XOD	Downregulate URAT1	Upregulate OAT1	[25]
Total saponins of *Dioscorea*	Inhibit activity of XOD	Downregulate URAT1	Upregulate OAT1 and OAT3	[26]
*Lagotis brevituba* Maxim. (LBM) extracts	Inhibit activity of XOD	Downregulate renal URAT1 and GLUT9	Upregulate OAT1	[27]
*P. sibircum* Laxm. alcohol extract 2.0/4.0/8.0 g/kg	Inhibit activity of XOD	Reduce GLUT9	Upregulate OAT1	[28]
*P. sibircum* Laxm. alcohol extract 8.0 g/kg	Inhibit activity of XOD	Reduce URAT1 and GLUT9	Upregulate OAT1	[28]
